# Impact of Laparoscopic Liver Resection on Long‐Term Outcomes and Non‐Liver‐Related Mortality in Elderly Patients With Primary Hepatocellular Carcinoma: A Multicenter Propensity Score‐Matched Analysis

**DOI:** 10.1002/ags3.70306

**Published:** 2026-11-02

**Authors:** Yukio Tokumitsu, Hidenori Takahashi, Yuki Nakagami, Toru Hisaka, Hiromitsu Hayashi, Tomoharu Yoshizumi, Norifumi Harimoto, Takao Ide, Yoichi Yamasaki, Atsushi Miyoshi, Yuichi Endo, Tamotsu Kuroki, Atsushi Nanashima, Masahiko Sakoda, Tohru Utsunomiya, Kohji Okamoto, Masatoshi Kajiwara, Mitsuhisa Takatsuki, Toru Beppu, Susumu Eguchi

**Affiliations:** ^1^ Kyushu Study Group of Liver Surgery Nagasaki University Graduate School of Biomedical Sciences Nagasaki Japan; ^2^ Department of Gastroenterological, Breast and Endocrine Surgery Yamaguchi University Graduate School of Medicine Ube Japan; ^3^ Health Data Science Lab, Faculty of Data Science Shimonoseki City University Shimonoseki Japan; ^4^ Division of Hepatobiliary and Pancreatic Surgery, Department of Surgery Kurume University Kurume Japan; ^5^ Department of Gastroenterological Surgery, Graduate School of Life Sciences Kumamoto University Kumamoto Japan; ^6^ Department of Surgery and Science, Graduate School of Medical Sciences Kyushu University Fukuoka Japan; ^7^ Department of Hepato‐Biliary‐Pancreatic Surgery, Clinical Research Institute National Hospital Organization Kyushu Medical Center Fukuoka Japan; ^8^ Department of Surgery Saga University Faculty of Medicine Saga Japan; ^9^ Department of Digestive Surgery Kagoshima University Graduate School of Medical and Dental Sciences Kagoshima Japan; ^10^ Department of Surgery Saga‐Ken Medical Centre Koseikan Saga Japan; ^11^ Department of Gastroenterological and Pediatric Surgery Oita University Faculty of Medicine Graduate School of Medicine Yufu Japan; ^12^ Clinical Research Center and Department of Surgery National Hospital Organization Nagasaki Medical Center Ōmura Japan; ^13^ Division of Hepato‐Biliary‐Pancreas Surgery, Department of Surgery University of Miyazaki Faculty of Medicine Miyazaki Japan; ^14^ Department of Surgery Kagoshima Kouseiren Hospital Kagoshima Japan; ^15^ Department of Surgery Oita Prefectural Hospital Oita Japan; ^16^ Department of Surgery, Gastroenterology and Hepatology Center Kitakyushu City Yahata Hospital Kitakyushu Japan; ^17^ Department of Gastroenterological Surgery, Faculty of Medicine Fukuoka University Fukuoka Japan; ^18^ Department of Digestive and General Surgery, Graduate School of Medicine University of the Ryukyus Ginowan Japan; ^19^ Department of Surgery Yamaga City Medical Center Yamaga Japan; ^20^ Department of Surgery Nagasaki University Graduate School of Biomedical Sciences Nagasaki Japan

**Keywords:** aged 80 and over, cause of death, hepatectomy, hepatocellular carcinoma, laparoscopy

## Abstract

**Background:**

Elderly patients with hepatocellular carcinoma (HCC) are increasingly undergoing hepatectomy. Although laparoscopic liver resection (LLR) has been shown to provide better short‐term outcomes than open liver resection (OLR), long‐term outcomes are generally considered comparable. However, the impact of surgical approach on cause‐specific death, particularly non‐liver‐related death, remains unclear in elderly patients.

**Methods:**

This multicenter retrospective study included 969 patients ≥ 65 years old who underwent curative hepatectomy for solitary primary HCC ≤ 5 cm in maximum diameter at 18 institutions between 2010 and 2018. Propensity score matching was performed to reduce selection bias. Short‐ and long‐term outcomes, including liver‐related and non‐liver‐related death, were compared between LLR and OLR. Subgroup analyses were conducted according to age group.

**Results:**

After matching, LLR was significantly associated with lower blood loss, fewer postoperative complications, and shorter hospital stay than OLR. In the entire cohort, overall survival did not differ between groups. Among non‐liver‐related deaths, respiratory disease‐related death was more frequent in the OLR group. Among patients ≥ 80 years old, the incidence of non‐liver‐related death excluding death from other cancers was significantly lower in the LLR group, even after propensity score matching.

**Conclusions:**

LLR may reduce the risk of non‐liver‐related death excluding death from other cancers in patients ≥ 80 years old with solitary HCC ≤ 5 cm in maximum diameter. When determining surgical strategies in elderly patients, it is important to consider long‐term systemic outcomes in addition to oncological factors. In patients for whom LLR is technically feasible, its minimally invasive nature may provide clinical benefits.

## Graphical abstract

In this multicenter, propensity score–matched study of elderly patients with primary hepatocellular carcinoma, we compared long‐term cause‐specific mortality between laparoscopic (LLR) and open liver resection (OLR) using a competing‐risks framework. Among patients aged ≥ 80 years, LLR was associated with a lower 5‐year cumulative incidence of death from other non‐cancer causes than OLR (7.9% vs. 16.2%; *p* = 0.046), whereas liver‐related and other‐cancer mortality did not differ. These exploratory findings suggest that the minimally invasive nature of LLR may help preserve long‐term systemic outcomes in very elderly patients for whom it is technically feasible.

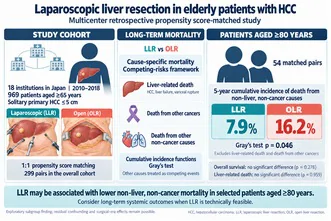

## 1 Introduction

Hepatocellular carcinoma (HCC) is the most common primary malignancy of the liver, and hepatectomy remains a mainstay of curative treatment [1, 2, 3]. In recent years, the age at diagnosis of HCC has increased in Japan in parallel with the aging of the population, resulting in a growing number of elderly patients undergoing hepatectomy [4]. Traditionally, hepatectomy in elderly patients has been considered with caution because of the invasiveness of the intervention. However, recent advances in perioperative management have enabled hepatectomy to be performed more safely in this population [5].

On the other hand, large‐scale database studies in Japan have demonstrated that long‐term outcomes after hepatectomy for HCC remain poorer in elderly patients than in younger patients, with non‐cancer‐related death representing a major contributing factor [6]. Prognosis in elderly patients is thus influenced not only by HCC‐related death but also by non‐liver‐related death associated with comorbid conditions.

In recent years, advances in surgical techniques and instruments have allowed widespread adoption of LLR [7]. Compared with OLR, LLR has been associated with improved short‐term outcomes, including reduced blood loss and fewer postoperative complications [8, 9, 10]. In addition, LLR has been reported to reduce the risk of complications such as ascites in patients with underlying chronic liver disease [11, 12]. These advantages of minimally invasive surgery have also been observed in elderly patients [13, 14]. Beyond hepatectomy, the short‐term benefits of laparoscopic surgery in elderly patients have likewise been reported in other gastrointestinal malignancies [15]. Further, several studies have demonstrated comparable long‐term oncological outcomes between LLR and OLR [9, 16]. However, most such studies have focused on overall or recurrence‐free survival. Limited attention has therefore been paid to cause‐specific death and non‐liver‐related death in particular, which is a clinically important outcome in elderly patients. The present study therefore aimed to compare short‐ and long‐term outcomes between LLR and OLR in elderly patients with primary HCC using a multicenter dataset, with a particular focus on cause‐specific death.

## 2 Materials and Methods

### 2.1 Patients

This multicenter retrospective study was conducted by the Kyushu Study Group of Liver Surgery in Japan and was registered with the University Hospital Medical Information Network (UMIN) Clinical Trials Registry (UMIN000057870). Consecutive patients with HCC who underwent hepatectomy at 18 participating institutions between January 2010 and December 2018 were screened. Participating institutions comprised Yamaguchi University, Kurume University, Kumamoto University, Kyushu University, National Hospital Organization Kyushu Medical Center, Saga University, Kagoshima University, Saga‐Ken Medical Centre Koseikan, Nagasaki University, Oita University, National Hospital Organization Nagasaki Medical Center, University of Miyazaki, Kagoshima Kouseiren Hospital, Oita Prefectural Hospital, Kitakyushu City Yahata Hospital, Fukuoka University, University of the Ryukyus, and Yamaga City Medical Center.

Eligible patients met the following criteria: (1) age ≥ 65 years; (2) solitary HCC ≤ 5 cm in maximum diameter; and (3) pathologically confirmed HCC. Patients could be any sex. Exclusion criteria were as follows: (1) active concomitant malignancies; (2) prior treatment for HCC, including liver resection, percutaneous local ablative therapy, transarterial (chemo)embolization, or radiotherapy; (3) tumor thrombus affecting portal or hepatic veins; (4) lymph node metastasis; (5) distant metastasis; (6) combined procedures such as vascular resection and reconstruction, tumor thrombectomy, biliary reconstruction, simultaneous resection of other organs (except the gallbladder), or intraoperative local ablative therapy; (7) multiple liver lesions; (8) laparoscopic‐assisted open liver resection, hand‐assisted laparoscopic surgery, thoracoscopic surgery, thoracolaparotomy, or robotic‐assisted surgery; or (9) incomplete clinical data.

The study protocol was approved by the institutional review boards of Yamaguchi University Hospital (approval no. H2024‐141) and each participating institution. Clinical data were retrospectively collected and centrally analyzed at Yamaguchi University.

The Charlson Comorbidity Index (CCI) was used to assess comorbidities. The non‐age‐adjusted CCI was applied because all patients were ≥ 65 years old and subgroup analyses were performed according to age strata. This approach avoided overestimation of age‐related effects and allowed evaluation of comorbidities independent of age.

### 2.2 Study Design

This study compared short‐ and long‐term outcomes between LLR and OLR using propensity score matching (PSM).

Cause of death was ascertained at each participating institution from the medical records and follow‐up (prognostic) surveys, and was recorded using a common, prespecified list applied uniformly across all institutions; central adjudication of individual cause‐of‐death assignments was not performed. Death events were classified into two categories: (1) liver‐related death, including HCC‐related death, liver failure‐related death, and variceal rupture; and (2) non‐liver‐related death. Because HCC survivors, particularly elderly patients and long‐term survivors, are at non‐negligible risk of developing second primary malignancies [17, 18], We further divided non‐liver‐related death into death from other cancers and death from other non‐cancer causes; the former reflects a patient's independent oncologic risk unrelated to the surgical approach, whereas the latter more directly reflects the systemic physiological burden of surgery. Liver‐related death, death from other cancers, and death from other non‐cancer causes were treated as competing events.

To evaluate the impact of age among elderly patients, subgroup analyses were performed by stratifying patients into four age groups: 65–69 years; 70–74 years; 75–79 years; and ≥ 80 years.

### 2.3 Statistical Analysis

Categorical variables were compared using the *χ* ^2^ test or Fisher's exact test, as appropriate, and continuous variables were compared using the Mann–Whitney U test.

To reduce selection bias, PSM was performed using a 1:1 nearest‐neighbor matching algorithm with a caliper width of 0.2 of the standard deviation of the logit of the propensity score.

The variables included in propensity score models were age, sex, body mass index (BMI), Eastern Cooperative Oncology Group Performance Status (ECOG‐PS), American Society of Anesthesiologists (ASA) physical status, CCI, hepatitis B surface antigen, hepatitis C virus antibody, presence of liver cirrhosis, Child–Pugh score, tumor size, tumor location (posterosuperior vs. anterolateral segments), proximity to major vessels (< 1 cm), previous abdominal or liver surgery, and resected area. In subgroup analyses, matching was performed using the same variables, excluding age.

The median follow‐up period was estimated using the reverse Kaplan–Meier method. Overall survival was estimated using the Kaplan–Meier method and compared between groups using log‐rank testing. Cause‐specific deaths (including liver‐related death, death from other cancers, and death from other non‐cancer causes) were analyzed using the cumulative incidence function, with remaining causes of death treated as competing events, and compared between groups using Gray's test. All statistical analyses were performed using JMP version 19.0 (SAS Institute Japan) except for Gray's tests, which were performed using R software version 4.5.2 (R Foundation for Statistical Computing, Vienna, Austria). Values of *p* < 0.05 were considered statistically significant.

## 3 Results

### 3.1 Patient Selection and Characteristics

A total of 1108 patients with HCC who underwent hepatectomy at the 18 participating institutions between January 2010 and December 2018 and met the inclusion criteria were initially identified. Among these, 139 patients were excluded for the following reasons: previous liver resection for HCC (*n* = 5); portal or hepatic vein tumor thrombus (*n* = 15); lymph node metastasis (*n* = 1); distant metastasis (*n* = 1); intraoperative local ablative therapy (*n* = 3); simultaneous distal gastrectomy (*n* = 1); laparoscopic‐assisted open liver resection through a short laparotomy (*n* = 74); hand‐assisted laparoscopic surgery (*n* = 4); thoracoscopic surgery (*n* = 1); thoracolaparotomy (*n* = 13); and missing medical records or laboratory data (*n* = 21). Finally, 969 patients were included in the present study (Figure 1).

**FIGURE 1:**
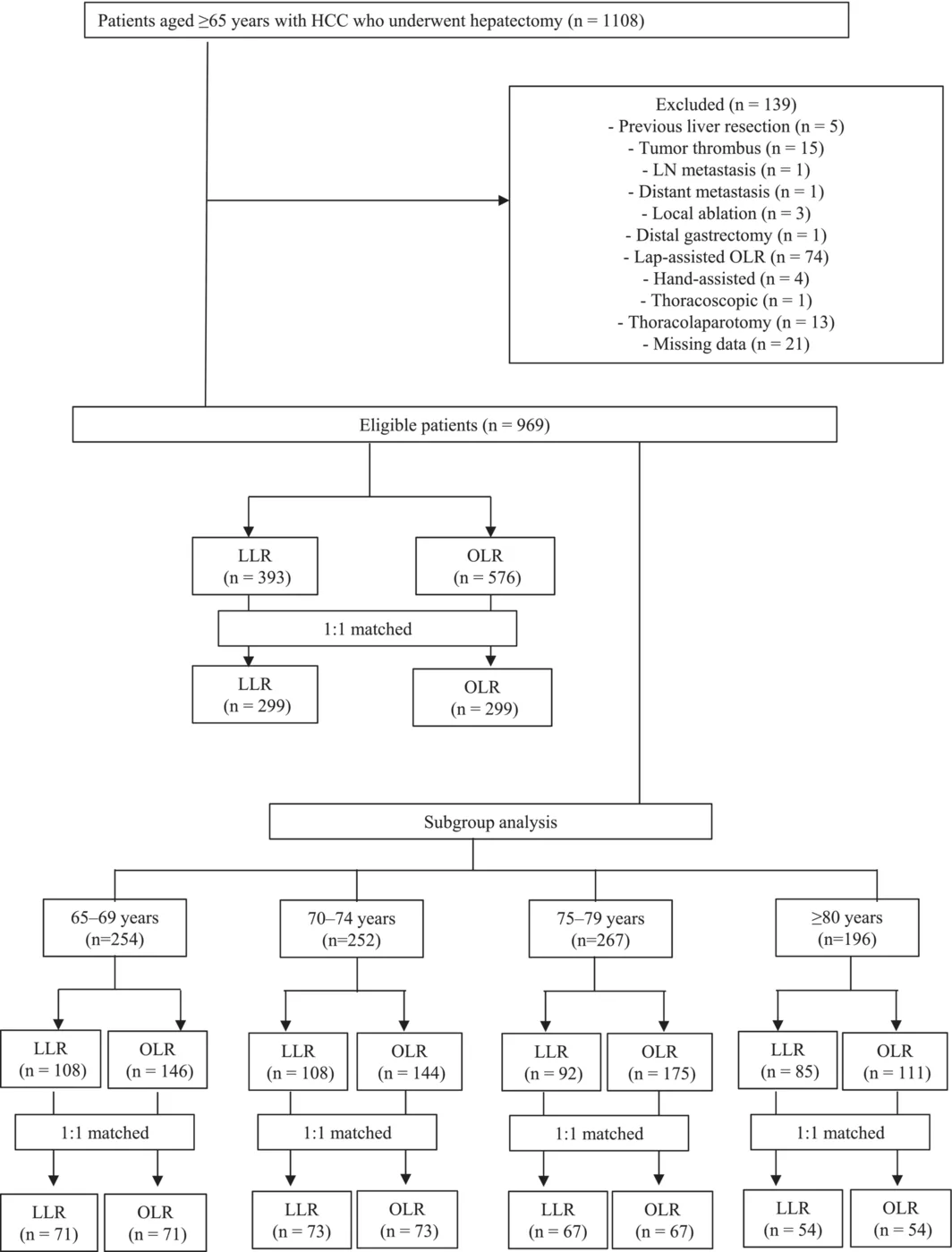
Patient flow diagram. A total of 1108 patients ≥ 65 years old with hepatocellular carcinoma (HCC) who underwent hepatectomy at 18 participating institutions between January 2010 and December 2018 were initially screened. Application of the exclusion criteria removed 139 patients, leaving 969 eligible patients (393 in the laparoscopic liver resection [LLR] group and 576 in the open liver resection [OLR] group). One‐to‐one propensity score matching yielded 299 matched pairs for analysis of the entire cohort. For subgroup analyses, patients were stratified into four age categories (65–69, 70–74, 75–79, and ≥ 80 years), and propensity score matching was performed within each subgroup. Detailed sample sizes before and after matching are shown for each subgroup.

Patient characteristics are summarized in Table 1. Before PSM, the LLR group showed smaller tumors, a higher proportion of tumors located in the anterolateral segments, fewer tumors in proximity to major vessels, and a lower prevalence of liver cirrhosis compared with the OLR group (*p* < 0.001 each).

**TABLE 1:** Baseline characteristics of patients before and after propensity score matching.

		Total	Before matching			After matching		
			LLR (*n* = 393)	OLR (*n* = 576)	*p*	LLR (*n* = 299)	OLR (*n* = 299)	*p*
Age	Median [range]	74 [65–92]	73 [65–92]	74 [65–91]	0.705	73 [65–91]	75 [65–92]	0.495
Sex	Male	654	253	401	0.087	193	194	0.932
	Female	315	140	175		106	105	
BMI	< 25 kg/m^2^	680	277	403	0.863	209	211	0.858
	≥ 25 kg/m^2^	289	116	173		90	88	
ECOG‐PS	0	748	309	439	0.380	228	229	0.923
	≥ 1	221	84	137		71	70	
ASA	≤ 2	883	365	518	0.114	277	279	0.749
	≥ 3	86	28	58		22	20	
CCI	≤ 2	494	218	276	0.299	161	153	0.512
	≥ 3	475	175	300		138	146	
HBsAg	(+)	109	47	62	0.563	39	32	0.376
	(−)	860	346	514		260	267	
HCV Ab	(+)	487	204	283	0.396	148	155	0.567
	(−)	482	189	293		151	144	
LC	(+)	299	147	152	< 0.001	99	97	0.862
	(−)	670	246	424		200	202	
Child–Pugh score	5	764	296	468	0.055	226	223	0.836
	6	158	72	86		56	61	
	≥ 7	47	25	22		17	15	
Tumor diameter (cm)	≤ 3	651	303	348	< 0.001	217	212	0.650
	> 3	318	90	228		82	87	
Tumor location	AL	644	305	339	< 0.001	214	222	0.462
	PS	325	88	237		85	77	
Proximity to major vessels	(−)	835	366	469	< 0.001	273	267	0.407
	(+)	134	27	107		26	32	
History of upper abdominal surgery	(−)	792	327	465	0.327	247	240	0.462
	(+)	177	66	111		52	59	
Resected area	Wedge	430	260	170	< 0.001	167	161	0.501
	Segment	178	36	142		36	29	
	Section	267	90	177		89	104	
	2 Sections	94	7	87		7	5	

Abbreviations: AL, anterolateral; ASA, American Society of Anesthesiologists status; BMI, body mass index; CCI, Charlson Comorbidity Index; ECOG‐PS, Eastern Cooperative Oncology Group performance status; HBsAg, hepatitis B surface antigen; HCV Ab, hepatitis C virus antibody; LC, liver cirrhosis; LLR, laparoscopic liver resection; OLR, open liver resection; PS, posterosuperior.

After PSM, no significant differences were observed between the two groups in baseline characteristics, including age (73 vs. 75 years, *p* = 0.495), sex, BMI, ECOG‐PS, ASA physical status, CCI, liver function, tumor‐related factors, and surgical factors, indicating adequate balance between groups (*p* > 0.05 each).

In the matched cohort, the median follow‐up (reverse Kaplan–Meier) was consistently shorter in the LLR than the OLR group (87.2 vs. 102.2 months overall; group medians, 81.4–87.2 vs. 93.4–108.2 months; Table S1), reflecting the later adoption of LLR (median year of surgery, 2016 vs. 2014 overall; group medians, 2016–2017 vs. 2014; *p* < 0.001 for each).

### 3.2 Short‐Term Outcomes for the Entire Cohort

Short‐term outcomes are shown in Table 2.

**TABLE 2:** Comparison of short‐term outcomes between LLR and OLR before and after propensity score matching.

		Before matching			After matching		
		LLR (*n* = 393)	OLR (*n* = 576)	*p*‐value	LLR (*n* = 299)	OLR (*n* = 299)	*p*
Blood loss (mL)	Median [range]	67 [0–2933]	370 [0–3920]	< 0.001	67 [0–2933]	335 [0–3138]	< 0.001
Blood transfusion	*n* (%)	16 (4.07)	62 (10.76)	< 0.001	13 (4.35)	29 (9.70)	0.011
Operation time (min)	Median [range]	237 [67–722]	317 [61–774]	0.048	246 [65–722]	280 [61–774]	0.001
Margin (cm)	Median [range]	0.5 [0–7]	0.5 [0–12]	0.247	0.5 [0–7]	0.5 [0–12]	0.397
Postoperative complications							
Clavien–Dindo ≥ 2	*n* (%)	48 (12.21)	168 (29.17)	< 0.001	35 (13.71)	82 (29.91)	< 0.001
Mortality	*n* (%)	2 (0.51)	3 (0.52)	0.992	2 (0.67)	2 (0.67)	1.000
Postoperative hospital stay (days)	Median [range]	9 [4–106]	14 [4–164]	< 0.001	9 [4–106]	14 [4–398]	< 0.001

Abbreviations: LLR, laparoscopic liver resection; OLR, open liver resection.

Before matching, the LLR group displayed significantly lower intraoperative blood loss (67 vs. 370 mL, *p* < 0.001), a lower transfusion rate (4.07% vs. 10.76%, *p* < 0.001), shorter operative time (237 vs. 317 min, *p* = 0.048), fewer postoperative complications (Clavien–Dindo grade ≥ 2: 12.21% vs. 29.17%, *p* < 0.001), and shorter postoperative hospital stay (9 vs. 14 days, *p* < 0.001) than the OLR group.

After matching, these differences remained significant. The LLR group showed lower blood loss (67 vs. 335 mL, *p* < 0.001), a lower transfusion rate (4.35% vs. 9.70%, *p* = 0.011), shorter operative time (246 vs. 280 min, *p* = 0.001), fewer postoperative complications (13.71% vs. 29.91%, *p* < 0.001), and shorter hospital stay (9 vs. 14 days, *p* < 0.001). Postoperative mortality did not differ between the two groups (0.67% vs. 0.67%, *p* = 1.000).

### 3.3 Long‐Term Outcomes in the Entire Cohort

Long‐term outcomes are shown in Table 3 and Figure 2.

**TABLE 3:** Comparison of causes of death between LLR and OLR.

	LLR (*n* = 393) [65–69/70–74/75–79/≥ 80]	OLR (*n* = 576) [65–69/70–74/75–79/≥ 80]	*p*
Death	129 (32.8%) [25/34/37/33]	209 (36.3%) [45/44/66/54]	0.273
Liver‐related death	79 (20.1%) [17/25/20/17]	123 (21.4%) [27/29/42/25]	0.687
Hepatocellular carcinoma	60 (15.3%) [13/18/15/14]	108 (18.8%) [25/25/34/24]	0.168
Liver failure	16 (4.1%) [3/6/4/3]	10 (1.7%) [2/3/5/0]	0.041
Variceal rupture	2 (0.5%) [1/1/0/0]	1 (0.2%) [0/0/1/0]	0.570
Other	1 (0.3%) [0/0/1/0]	4 (0.7%) [0/1/2/1]	0.654
Non‐liver‐related death	50 (12.7%) [8/9/17/16]	86 (14.9%) [18/15/24/29]	0.326
Other cancers	21 (5.3%) [3/4/6/8]	15 (2.6%) [6/3/4/2]	0.037
Other non‐cancer causes	29 (7.4%) [5/5/11/8]	71 (12.3%) [12/12/20/27]	0.013
Pulmonary disease	7 (1.8%) [1/3/1/2]	26 (4.5%) [4/6/5/11]	0.029
Cardiovascular disease	3 (0.8%) [1/0/0/2]	12 (2.1%) [3/3/3/3]	0.118
Cerebrovascular disease	3 (0.8%) [1/0/2/0]	1 (0.2%) [0/0/0/1]	0.310
Renal disease	1 (0.3%) [0/0/1/0]	3 (0.5%) [0/1/0/2]	0.651
Senility	0 (0.0%) [0/0/0/0]	3 (0.5%) [0/1/2/0]	0.276
Other	3 (0.8%) [1/0/1/1]	10 (1.7%) [2/0/3/5]	0.260
Details unknown	12 (3.1%) [1/2/6/3]	16 (2.8%) [3/1/7/5]	0.846

Abbreviations: LLR, laparoscopic liver resection; OLR, open liver resection.

**FIGURE 2:**
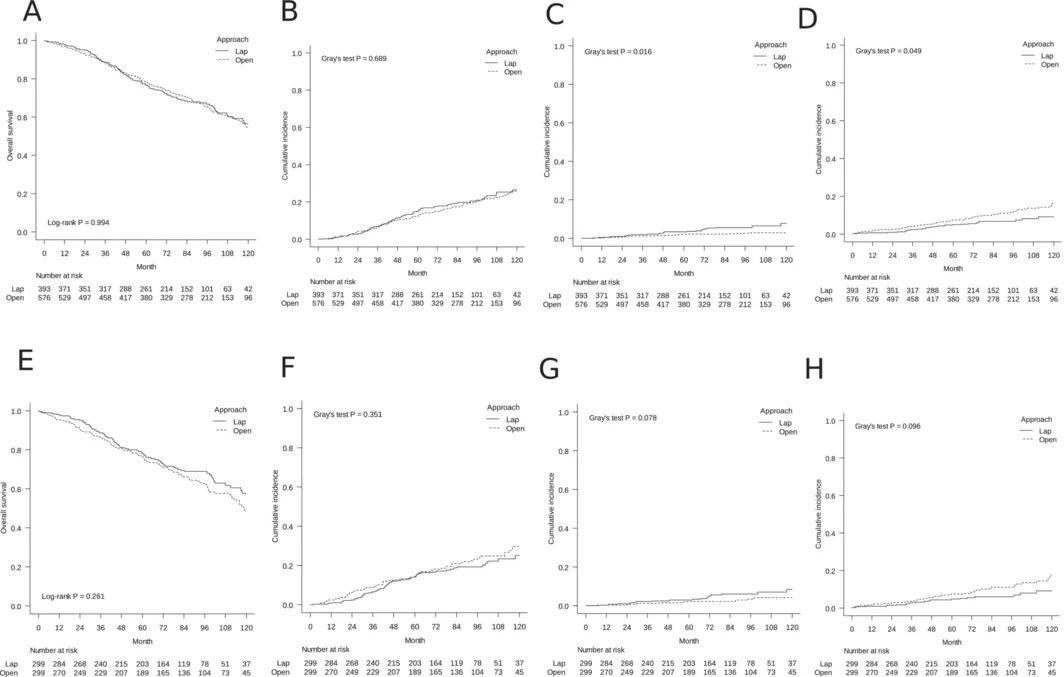
Long‐term survival outcomes in the entire cohort before and after propensity score matching. (A–D) Outcomes before matching. (E–H) Corresponding outcomes after matching. (A, E) Overall survival estimated by the Kaplan–Meier method and compared between LLR and OLR groups using log‐rank testing (A, *p* = 0.994; E, *p* = 0.261). (B–D, F–H) Cumulative incidence of cause‐specific death, with remaining causes of death treated as competing events, compared between groups using Gray's test: (B, F) liver‐related death (*p* = 0.689 and *p* = 0.351); (C, G) death from other cancers (*p* = 0.016 and *p* = 0.078); and (D, H) death from other non‐cancer causes (*p* = 0.049 and *p* = 0.096), before and after matching, respectively. LLR, laparoscopic liver resection; OLR, open liver resection.

The number of all‐cause deaths was 129 (32.8%) in the LLR group and 209 (36.3%) in the OLR group, showing no significant difference (*p* = 0.273). Overall survival did not differ significantly between groups either before (log‐rank *p* = 0.994) or after PSM (log‐rank *p* = 0.261) (Figure 2A,E).

Liver‐related death occurred in 79 patients (20.1%) and 123 patients (21.4%), respectively (*p* = 0.687), and HCC‐related death did not differ between groups (60 [15.3%] vs. 108 [18.8%], *p* = 0.168). Within liver‐related death, liver failure‐related death was significantly more frequent in the LLR group (16 [4.1%] vs. 10 [1.7%], *p* = 0.041). The cumulative incidence of liver‐related death did not differ significantly between groups before or after matching (Gray's test *p* = 0.689 and *p* = 0.351, respectively; Figure 2B,F).

The overall frequency of non‐liver‐related death did not differ significantly between groups (50 [12.7%] vs. 86 [14.9%], *p* = 0.326). However, death from other cancers was significantly more frequent in the LLR group (21 [5.3%] vs. 15 [2.6%], *p* = 0.037), whereas death from other non‐cancer causes was significantly more frequent in the OLR group (29 [7.4%] vs. 71 [12.3%], *p* = 0.013). Among deaths from other non‐cancer causes, respiratory disease‐related death was significantly more frequent in the OLR group (7 [1.8%] vs. 26 [4.5%], *p* = 0.029). In the analysis of cumulative incidence, death from other cancers was significantly higher in the LLR group before matching (Gray's test *p* = 0.016), whereas death from other non‐cancer causes was significantly higher in the OLR group before matching (Gray's test *p* = 0.049). After matching, neither difference reached the level of statistical significance (Gray's test *p* = 0.078 and *p* = 0.096, respectively) (Figure 2C,D,G,H).

### 3.4 Subgroup Analysis

#### 3.4.1 Patients Aged 65–69 Years

Patient characteristics are shown in Table S2. After matching, no significant differences were observed between groups. Short‐term outcomes (Table S3) showed significantly lower intraoperative blood loss, fewer postoperative complications, and shorter hospital stay in the LLR group compared with the OLR group, both before and after matching (*p* < 0.01 each).

Long‐term outcomes (Figure S1) showed no significant differences between groups. Overall survival did not differ significantly between groups (log‐rank *p* = 0.340 before PSM, *p* = 0.247 after PSM), nor did the cumulative incidences of liver‐related death (Gray's test *p* = 0.783 and *p* = 0.724), death from other cancers (*p* = 0.612 and *p* = 0.368), or death from other non‐cancer causes (*p* = 0.363 and *p* = 0.106) before and after PSM, respectively.

#### 3.4.2 Patients Aged 70–74 Years

Patient characteristics are shown in Table S4, with no significant differences after matching. Short‐term outcomes (Table S5) demonstrated significantly lower blood loss and fewer postoperative complications in the LLR group, both before and after PSM (*p* < 0.05 each).

No significant differences in long‐term outcomes were observed (Figure S2). Overall survival did not differ between groups (log‐rank *p* = 0.468 before and *p* = 0.955 after PSM), nor did the cumulative incidence of liver‐related death (Gray's test *p* = 0.343 and *p* = 0.519), death from other cancers (*p* = 0.375 and *p* = 0.509), or death from other non‐cancer causes (*p* = 0.378 and *p* = 0.517) before and after PSM, respectively.

#### 3.4.3 Patients Aged 75–79 Years

Patient characteristics are shown in Table S6, with balanced baseline factors after matching. Short‐term outcomes (Table S7) showed significantly lower blood loss and shorter hospital stay in the LLR group, both before and after PSM (*p* < 0.05 each).

No significant differences in long‐term outcomes were observed (Figure S3). Overall survival did not differ between groups (log‐rank *p* = 0.200 before and *p* = 0.421 after PSM), nor did the cumulative incidences of liver‐related death (Gray's test *p* = 0.867 and *p* = 0.181), death from other cancers (*p* = 0.057 and *p* = 0.517), or death from other non‐cancer causes (*p* = 0.562 and *p* = 0.855) before and after PSM, respectively.

#### 3.4.4 Patients Aged ≥ 80 Years

Patient characteristics are shown in Table 4, with no significant differences after matching. In the matched cohort, the median follow‐up (reverse Kaplan–Meier) was 81.4 months in the LLR group and 93.4 months in the OLR group (*p* = 0.268; Table S1). In terms of short‐term outcomes (Table S8), blood loss was significantly lower and hospital stay was significantly shorter in the LLR group compared with the OLR group, both before and after PSM (*p* < 0.01 each).

**TABLE 4:** Baseline characteristics of patients aged ≥ 80 years before and after propensity score matching.

		Total	Before matching			After matching		
			LLR (*n* = 85)	OLR (*n* = 111)	*p*	LLR (*n* = 54)	OLR (*n* = 54)	*p*
Sex	Male	128	51	77	0.173	34	36	0.687
	Female	68	34	34		20	18	
BMI	< 25 kg/m^2^	152	62	90	0.176	41	44	0.481
	≥ 25 kg/m^2^	44	23	21		13	10	
ECOG‐PS	0	137	63	74	0.260	38	35	0.537
	≥ 1	59	22	37		16	19	
ASA	≤ 2	177	80	97	0.115	50	47	0.340
	≥ 3	19	5	14		4	7	
CCI	≤ 2	99	34	65	0.010	26	26	1.000
	≥ 3	97	51	46		28	28	
HBsAg	(+)	14	10	4	0.028	2	2	1.000
	(−)	182	75	107		52	52	
HCV Ab	(+)	102	43	59	0.722	27	31	0.440
	(−)	94	42	52		27	23	
LC	(+)	49	29	20	0.010	13	13	1.000
	(−)	147	56	91		41	41	
Child–Pugh score	5	152	65	87	0.654	41	38	0.431
	6	40	19	21		12	16	
	≥ 7	4	1	3		1	0	
Tumor diameter (cm)	≤ 3	107	58	49	0.001	31	25	0.248
	> 3	89	27	62		23	29	
Tumor location	AL	135	64	71	0.090	38	41	0.515
	PS	61	21	40		16	13	
Proximity to major vessels	(−)	172	80	92	0.017	50	50	1.000
	(+)	24	5	19		4	4	
History of upper abdominal surgery	(−)	145	64	81	0.714	38	40	0.667
	(+)	51	21	30		16	14	
Resected area	Wedge	83	51	32	< 0.001	27	27	0.483
	Segment	35	8	27		7	4	
	Section	64	23	41		17	22	
	2 Sections	14	3	11		3	1	

Abbreviations: AL, anterolateral; ASA, American Society of Anesthesiologists status; BMI, body mass index; CCI, Charlson Comorbidity Index; ECOG‐PS, Eastern Cooperative Oncology Group performance status; HBsAg, hepatitis B surface antigen; HCV Ab, hepatitis C virus antibody; LC, liver cirrhosis; LLR, laparoscopic liver resection; OLR, open liver resection; PS, posterosuperior.

Long‐term outcomes (Figure 3) showed no significant differences in overall survival (log‐rank *p* = 0.427 before and *p* = 0.278 after PSM) or in the cumulative incidence of liver‐related death (Gray's test *p* = 0.875 and *p* = 0.959, respectively). Death from other cancers was significantly higher in the LLR group before PSM (Gray's test *p* = 0.014) but not after PSM (*p* = 0.266). However, in contrast to the younger age groups, Gray's test demonstrated that the incidence of death from other non‐cancer causes was significantly lower in the LLR group both before (*p* = 0.012) and after PSM (*p* = 0.046). The corresponding 5‐year cumulative incidences were 7.9% (LLR) versus 16.2% (OLR) (Table 5).

**FIGURE 3:**
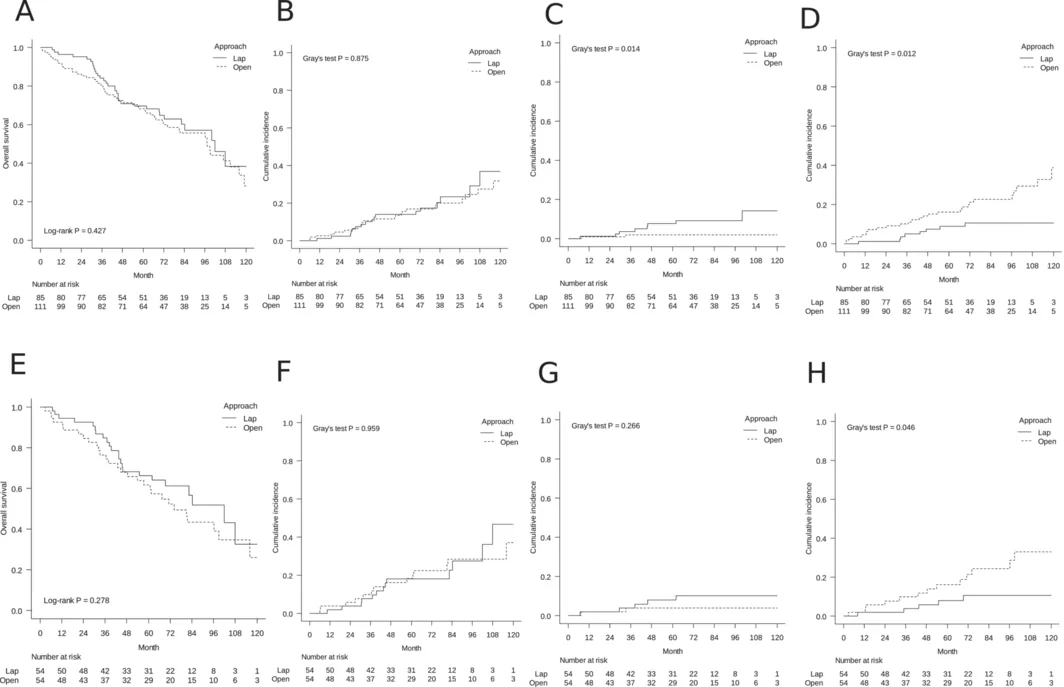
Long‐term survival outcomes in patients ≥ 80 years old before and after propensity score matching. (A–D) Outcomes before matching. (E–H) Corresponding outcomes after matching. (A, E) Overall survival estimated by the Kaplan–Meier method and compared between LLR and OLR groups using log‐rank testing (A, *p* = 0.427; E, *p* = 0.278). (B–D, F–H) Cumulative incidence of cause‐specific death, with remaining causes of death treated as competing events, compared between groups using Gray's test: (B, F) liver‐related death (*p* = 0.875 and *p* = 0.959); (C, G) death from other cancers (*p* = 0.014 and *p* = 0.266); and (D, H) death from other non‐cancer causes (*p* = 0.012 and *p* = 0.046), before and after matching, respectively. LLR, laparoscopic liver resection; OLR, open liver resection.

**TABLE 5:** Cause‐specific mortality in the propensity score–matched cohort of patients aged ≥ 80 years.

Cause of death	LLR (*n* = 54)		OLR (*n* = 54)		*p*
	No. of deaths	5‐year cumulative incidence, % (95% CI)	No. of deaths	5‐year cumulative incidence, % (95% CI)	
All‐cause death	23	33.8 (20.7–47.0)	31	38.4 (24.7–52.0)	0.278
Liver‐related death	13	17.9 (8.8–29.6)	16	18.3 (9.0–30.1)	0.959
Other‐cancer death	5	7.9 (2.5–17.4)	2	4.0 (0.7–12.0)	0.266
Other non‐cancer death	5	7.9 (2.5–17.4)	13	16.2 (7.5–27.7)	0.046

Abbreviations: CI, confidence interval; LLR, laparoscopic liver resection; OLR, open liver resection.

## 4 Discussion

This study demonstrated that LLR was associated with a significantly lower incidence of non‐liver‐related mortality, excluding death from other malignancies, compared with OLR in patients ≥ 80 years old, even after PSM. Although numerous studies have reported short‐term advantages of LLR [8, 9, 10, 11, 12, 13, 14], long‐term outcomes have generally been considered comparable between laparoscopic and open approaches [9, 16], and studies focusing on cause‐specific mortality remain scarce. Long‐term outcomes after hepatectomy in elderly patients are known to be inferior to those in younger patients, with an increase in non‐liver‐related mortality identified as a major contributing factor [6, 19]. The significance of the present study lies in its focus on non‐liver‐related mortality and its demonstration that laparoscopic surgery may influence long‐term outcomes in patients ≥ 80 years old, potentially through reductions in perioperative physiological stress and preservation of physical function. With advances in medical technology and healthcare systems leading to increased life expectancy in developed countries, the population ≥ 80 years old is increasingly recognized as an important target population for identifying subgroups with distinct treatment profiles and prognostic profiles [4, 20]. In this study, the benefit of LLR on non‐liver‐related mortality reached significance only in patients ≥ 80 years old, likely because too few non‐liver‐related events were seen in younger elderly subgroups to demonstrate a statistical difference, and because octogenarians have less physiological reserve, so the additional surgical stress of open hepatectomy is more likely to result in lasting systemic decline. Beyond perioperative physiological stress, host factors such as nutritional status have also been reported to influence long‐term outcomes after surgery in elderly patients, particularly octogenarians [21]. The persistence of this age‐related disadvantage over time is illustrated by Hatanaka et al. [4], who evaluated outcomes in patients with hepatocellular carcinoma ≥ 80 years old versus younger patients across different time periods and reported that, while survival rates improved significantly in the non‐elderly group in association with improved liver function and increased rates of curative treatment, no comparable improvement in prognosis was observed in the elderly group. This finding was attributed to the absence of an increase in the rate of curative treatment in the elderly group, along with an increase in non‐liver‐related mortality due to comorbidities. In the present study, among patients ≥ 80 years old, the LLR group demonstrated a significant reduction in non‐liver‐related mortality without an increase in liver‐related mortality compared with the OLR group. These findings suggest that it is important to select the surgical approach from a comprehensive perspective that encompasses not only oncological radicality but also the risk of non‐liver‐related mortality, and that LLR may represent a favorable option in this regard.

Regarding the breakdown of non‐liver‐related causes of death, respiratory disease‐related mortality was significantly more frequent in the OLR group in the present study. Although LLR has been reported to reduce perioperative respiratory complications [13, 14], studies demonstrating an association with long‐term respiratory mortality remain scarce. The musculature of the abdominal wall plays an important role in respiratory dynamics, particularly in terms of diaphragmatic function [22], and open surgery is known to restrict diaphragmatic excursion due to extensive muscle incision. In hepatectomy, transverse incisions such as the Benz incision or J‐shaped incision are frequently employed [23, 24], and damage to the transversus abdominis muscle, which is continuous with the diaphragm, may adversely affect respiratory function. In contrast, laparoscopic surgery minimizes injury to the abdominal wall musculature, potentially suppressing restriction of diaphragmatic excursion and the development of restrictive ventilatory impairment, thereby contributing to the long‐term preservation of respiratory function [25]. Further, laparoscopic surgery has been shown to potentially reduce long‐term respiratory mortality even in early gastric cancer surgery [26], supporting the utility of minimally invasive approaches in upper abdominal surgery.

In the present study, a substantial number of deaths attributable to causes other than respiratory disease were also observed, and the above‐mentioned mechanism alone cannot fully account for all non‐liver‐related mortality. However, long‐term declines in respiratory function are reportedly associated with impaired cardiac function [27], skeletal muscle dysfunction, and frailty [28], and the concept of inter‐organ crosstalk has become widely accepted in recent years. In the present study, cardiac disease was the second‐most common cause of death in the OLR group after respiratory disease, and an indirect contribution mediated by postoperative decline in respiratory function following open hepatectomy cannot be excluded. Conceivably, respiratory declines may lead to reduced exercise tolerance and progression of frailty, thus influencing long‐term non‐liver‐related mortality risk. From the perspective of such inter‐organ interactions, minimally invasive surgery may contribute to improved long‐term outcomes through the preservation of systemic physical function.

Before matching, both liver failure‐related death and death from other cancers were more frequent in the LLR group, likely reflecting baseline selection rather than the surgical approach. LLR patients more often had cirrhosis and impaired hepatic reserve, conferring a higher long‐term risk of liver failure, and more often had smaller, earlier‐stage tumors, whose better prognosis and longer survival increased the opportunity to die from competing non‐hepatic malignancies. Consistent with this, both differences were no longer significant after matching balanced hepatic reserve and tumor factors.

It should be noted; however, that while the present results suggest the potential benefits of LLR, not all patients are candidates for this approach. OLR is more appropriate in some cases, depending on the location and extent of the tumor as well as the technical complexity of the procedure. The findings of this study should therefore be interpreted as demonstrating that, among patients for whom LLR is technically feasible, the minimally invasive nature of the intervention may contribute to the improvements in long‐term systemic outcomes.

This study has several limitations. First, as a retrospective study, the possibility of selection bias cannot be entirely excluded. Although PSM was employed to adjust for confounding factors, the influence of unknown confounders may persist. In particular, LLR was performed in significantly later years than OLR, and this imbalance persisted after matching; a contribution of surgical era to the observed differences cannot be excluded. Second, because postoperative assessments of respiratory function and frailty were not directly measured, the underlying mechanisms cannot be definitively established. Prospective studies incorporating spirometry, diaphragmatic ultrasonography, and validated frailty assessments would be needed to substantiate this mechanism. Third, cause of death was assigned at each institution without central adjudication, and a certain proportion of non‐liver‐related deaths remained of undetermined cause. Misclassification of these events could have biased comparisons of cause‐specific mortality in either direction, and the present findings should therefore be interpreted within these constraints. Fourth, in the present analysis, death from other cancers was somewhat more frequent in the LLR group. Because the cumulative incidence of a given cause of death is influenced not only by its own rate but also by the rates of competing events, this higher incidence of death from other cancers in the LLR group may have mechanically lowered the cumulative incidence of death from other non‐cancer causes in the same group. As a result, we cannot rule out that this competing relationship partly contributed to the lower incidence of death from other non‐cancer causes observed with LLR in patients ≥ 80 years old. Given the modest statistical significance and the multiple subgroup comparisons performed, this finding should be regarded as exploratory and hypothesis‐generating, and warrants confirmation in prospective studies.

In conclusion, the present study suggests that LLR may reduce the risk of non‐liver‐related mortality, excluding death from other malignancies, among patients ≥ 80 years old with a solitary tumor measuring ≤ 5 cm in maximum diameter. When considering surgical indications for elderly patients, it is important to take into account not only oncological factors but also long‐term systemic outcomes. Laparoscopic surgery may thus offer meaningful advantages in cases where it is technically feasible.

## Author Contributions

**Yukio Tokumitsu:** conceptualization, writing – original draft, writing – review and editing, investigation, visualization, software. **Hidenori Takahashi:** conceptualization, methodology, writing – review and editing. **Yuki Nakagami:** validation, data curation, formal analysis, software. **Toru Hisaka:** investigation, methodology, resources. **Hiromitsu Hayashi:** methodology, investigation, resources. **Tomoharu Yoshizumi:** methodology, investigation, project administration. **Norifumi Harimoto:** methodology, investigation, resources. **Takao Ide:** methodology, investigation, resources. **Yoichi Yamasaki:** investigation. **Atsushi Miyoshi:** investigation, resources, conceptualization. **Yuichi Endo:** conceptualization, investigation, resources. **Tamotsu Kuroki:** investigation, resources. **Atsushi Nanashima:** conceptualization, investigation, resources, methodology. **Masahiko Sakoda:** investigation, resources. **Tohru Utsunomiya:** conceptualization, investigation, resources, methodology. **Kohji Okamoto:** conceptualization, investigation. **Masatoshi Kajiwara:** investigation, resources. **Mitsuhisa Takatsuki:** investigation, resources. **Toru Beppu:** conceptualization, investigation, supervision. **Susumu Eguchi:** conceptualization, investigation, supervision, project administration, writing – review and editing.

## Funding

The authors have nothing to report.

## Ethics Statement

This study was approved by the institutional review board of Yamaguchi University Hospital (approval no. H2024‐141). All informed consent was obtained from the patients.

## Conflicts of Interest

Susumu Eguchi is an editorial board member of Annals of Gastroenterological Surgery. Tomoharu Yoshizumi is an associate editor member of Annals of Gastroenterological Surgery.

## Supporting information


**Figure S1:** Long‐term survival outcomes in patients aged 65–69 years before and after propensity score matching. (A–D) Outcomes before matching. (E–H) Corresponding outcomes after matching. (A, E) Overall survival estimated by the Kaplan–Meier method and compared between LLR and OLR groups using log‐rank testing (A, *p* = 0.340; E, *p* = 0.247). (B–D, F–H) Cumulative incidence of cause‐specific death, with remaining causes of death treated as competing events, compared between groups using Gray's test: (B, F) liver‐related death (*p* = 0.783 and *p* = 0.724); (C, G) death from other cancers (*p* = 0.612 and *p* = 0.368); and (D, H) death from other non‐cancer causes (*p* = 0.363 and *p* = 0.106), before and after matching, respectively. LLR, laparoscopic liver resection; OLR, open liver resection.
**Figure S2:** Long‐term survival outcomes in patients aged 70–74 years before and after propensity score matching. (A–D) Outcomes before matching. (E–H) Corresponding outcomes after matching. (A, E) Overall survival estimated by the Kaplan–Meier method and compared between LLR and OLR groups using log‐rank testing (A, *p* = 0.468; E, *p* = 0.955). (B–D, F–H) Cumulative incidence of cause‐specific death, with remaining causes of death treated as competing events, compared between groups using Gray's test: (B, F) liver‐related death (*p* = 0.343 and *p* = 0.519); (C, G) death from other cancers (*p* = 0.375 and *p* = 0.509); and (D, H) death from other non‐cancer causes (*p* = 0.378 and *p* = 0.517), before and after matching, respectively. LLR, laparoscopic liver resection; OLR, open liver resection.
**Figure S3:** Long‐term survival outcomes in patients aged 75–79 years before and after propensity score matching. (A–D) Outcomes before matching. (E–H) Corresponding outcomes after matching. (A, E) Overall survival estimated by the Kaplan–Meier method and compared between LLR and OLR groups using log‐rank testing (A, *p* = 0.200; E, *p* = 0.421). (B–D, F–H) Cumulative incidence of cause‐specific death, with remaining causes of death treated as competing events, compared between groups using Gray's test: (B, F) liver‐related death (*p* = 0.867 and *p* = 0.181); (C, G) death from other cancers (*p* = 0.057 and *p* = 0.517); and (D, H) death from other non‐cancer causes (*p* = 0.562 and *p* = 0.855), before and after matching, respectively. LLR, laparoscopic liver resection; OLR, open liver resection.
**Table S1:** Median follow‐up period by age group before and after propensity score matching.
**Table S2:** Baseline characteristics of patients aged 65–69 years before and after propensity score matching.
**Table S3:** Comparison of short‐term outcomes between LLR and OLR in patients aged 65–69 years before and after propensity score matching.
**Table S4:** Baseline characteristics of patients aged 70–74 years before and after propensity score matching.
**Table S5:** Comparison of short‐term outcomes between LLR and OLR in patients aged 70–74 years before and after propensity score matching.
**Table S6:** Baseline characteristics of patients aged 75–79 years before and after propensity score matching.
**Table S7:** Comparison of short‐term outcomes between LLR and OLR in patients aged 75–79 years before and after propensity score matching.
**Table S8:** Comparison of short‐term outcomes between LLR and OLR in patients aged ≥ 80 years before and after propensity score matching.

## Data Availability

The data that support the findings of this study are available from the corresponding author upon reasonable request.
