# Supplementary material for: Impact of Laparoscopic Liver Resection on Long‐Term Outcomes and Non‐Liver‐Related Mortality in Elderly Patients With Primary Hepatocellular Carcinoma: A Multicenter Propensity Score‐Matched Analysis
Source: Ann Gastroenterol Surg. 2026 Nov 2:10.1002/ags3.70306. Online ahead of print. doi: 10.1002/ags3.70306 (PMC13727762; doi:10.1002/ags3.70306)
Supplement: Supplementary file 1 [file AGS3-9999-0-s001.pdf]

Figure S1.

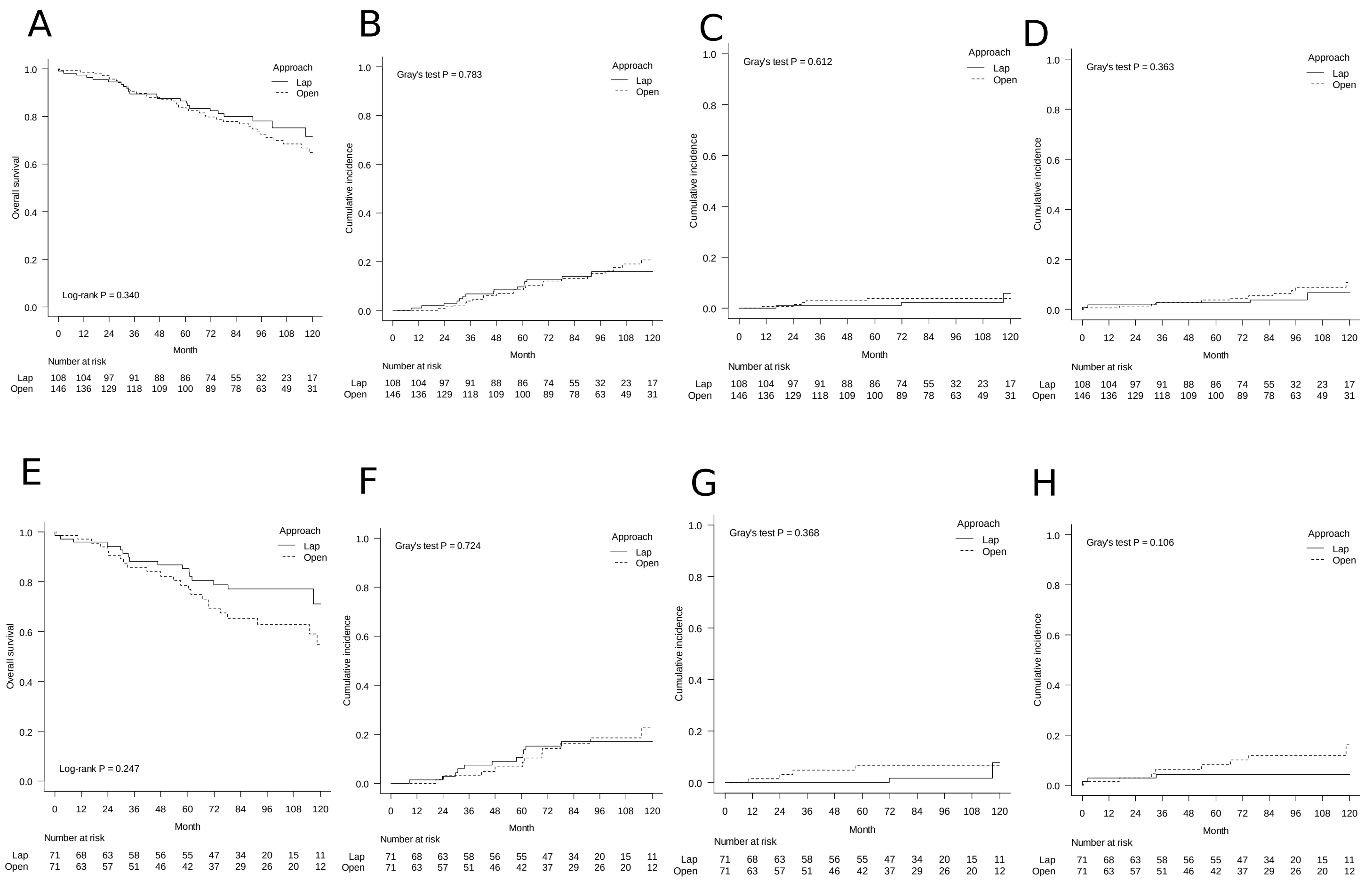
A
Approach
Lap
Open
Overall survival
Log-rank P = 0.340
Month
Number at risk
Lap 108 104 97 91 88 86 74 55 32 23 17
Open 146 136 129 118 109 100 89 78 63 49 31
B
Gray's test P = 0.783
Cumulative incidence
C
Gray's test P = 0.612
D
Gray's test P = 0.363
E
Log-rank P = 0.247
Lap 71 68 63 58 56 55 47 34 20 15 11
Open 71 63 57 51 46 42 37 29 26 20 12
F
Gray's test P = 0.724
G
Gray's test P = 0.368
H
Gray's test P = 0.106

Figure S2.

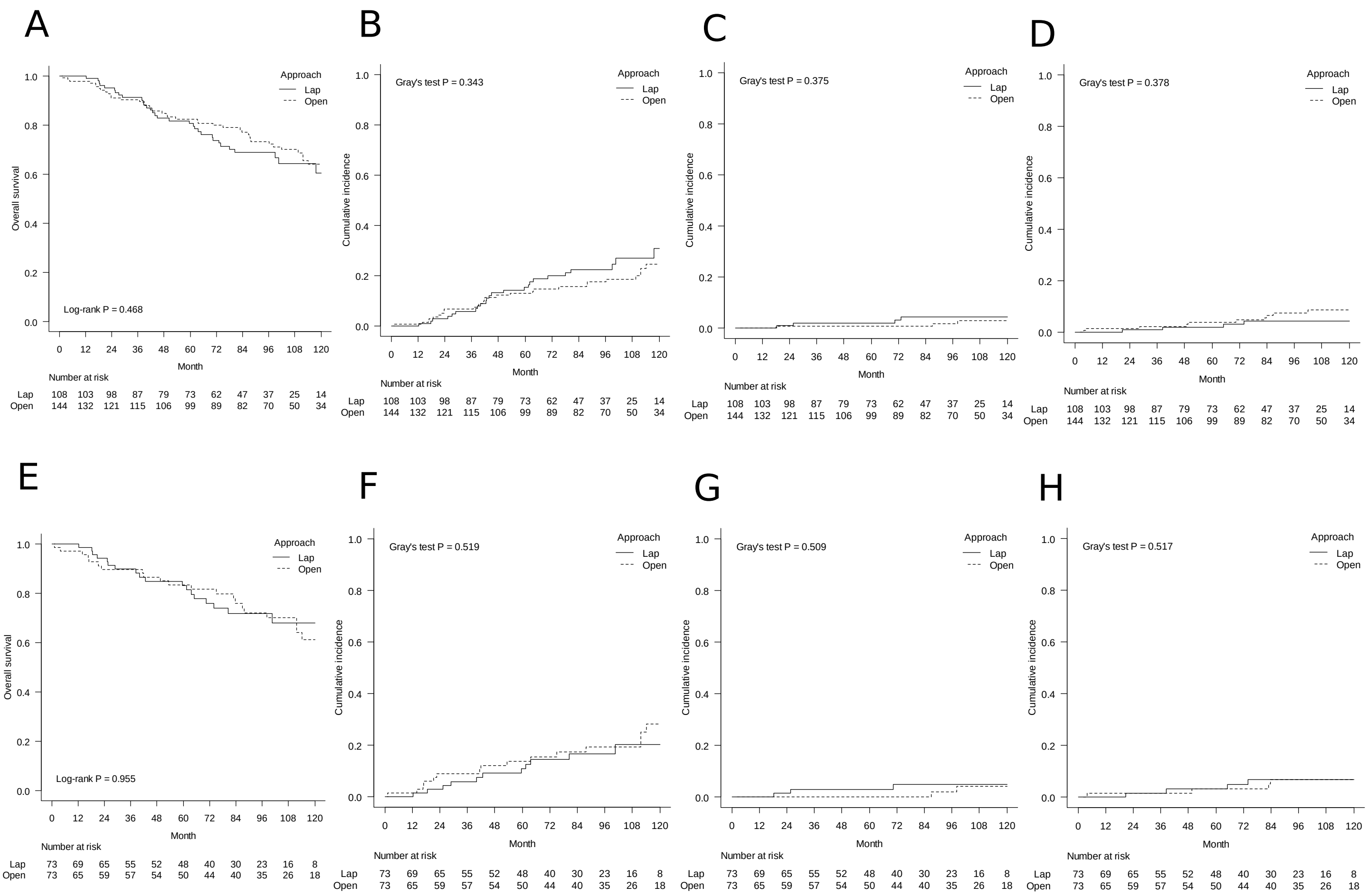
A
Approach
Lap
Open
Overall survival
Log-rank P = 0.468
Month
Number at risk
Lap 108 103 98 87 79 73 62 47 37 25 14
Open 144 132 121 115 106 99 89 82 70 50 34
B
Gray's test P = 0.343
Approach
Lap
Open
Cumulative incidence
Month
Number at risk
Lap 108 103 98 87 79 73 62 47 37 25 14
Open 144 132 121 115 106 99 89 82 70 50 34
C
Gray's test P = 0.375
Approach
Lap
Open
Cumulative incidence
Month
Number at risk
Lap 108 103 98 87 79 73 62 47 37 25 14
Open 144 132 121 115 106 99 89 82 70 50 34
D
Gray's test P = 0.378
Approach
Lap
Open
Cumulative incidence
Month
Number at risk
Lap 108 103 98 87 79 73 62 47 37 25 14
Open 144 132 121 115 106 99 89 82 70 50 34
E
Approach
Lap
Open
Overall survival
Log-rank P = 0.955
Month
Number at risk
Lap 73 69 65 55 52 48 40 30 23 16 8
Open 73 65 59 57 54 50 44 40 35 26 18
F
Gray's test P = 0.519
Approach
Lap
Open
Cumulative incidence
Month
Number at risk
Lap 73 69 65 55 52 48 40 30 23 16 8
Open 73 65 59 57 54 50 44 40 35 26 18
G
Gray's test P = 0.509
Approach
Lap
Open
Cumulative incidence
Month
Number at risk
Lap 73 69 65 55 52 48 40 30 23 16 8
Open 73 65 59 57 54 50 44 40 35 26 18
H
Gray's test P = 0.517
Approach
Lap
Open
Cumulative incidence
Month
Number at risk
Lap 73 69 65 55 52 48 40 30 23 16 8
Open 73 65 59 57 54 50 44 40 35 26 18

Figure S3.

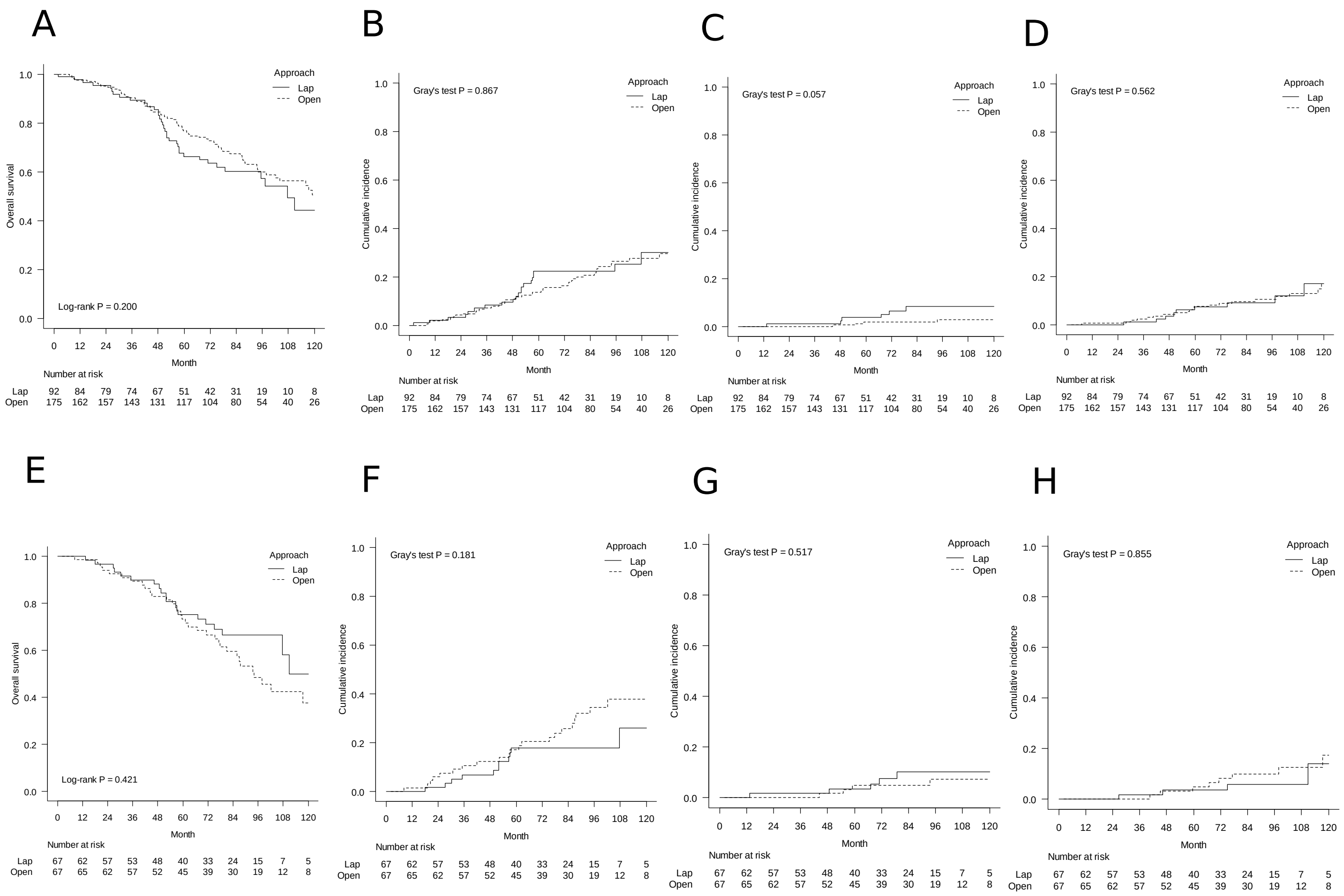
A
Approach
Lap
Open
Overall survival
Log-rank P = 0.200
Month
Number at risk
Lap 92 84 79 74 67 51 42 31 19 10 8
Open 175 162 157 143 131 117 104 80 54 40 26
B
Gray's test P = 0.867
Cumulative incidence
C
Gray's test P = 0.057
D
Gray's test P = 0.562
E
Log-rank P = 0.421
Lap 67 62 57 53 48 40 33 24 15 7 5
Open 67 65 62 57 52 45 39 30 19 12 8
F
Gray's test P = 0.181
G
Gray's test P = 0.517
H
Gray's test P = 0.855

Table S1. Median follow-up period by age group before and after propensity score matching

Age group	Before matching			After matching		
	LLR	OLR	P value	LLR	OLR	P value
65-69	87.7 [80.4–116.6]	106.0 [76.1–134.0]	0.060	87.2 [80.2–116.6]	102.2 [73.4–129.0]	0.400
70-74	92.4 [78.7–116.6]	107.7 [82.4–127.5]	0.042	86.1 [66.0–114.0]	108.2 [69.2–127.7]	0.005
75-79	86.4 [69.8–102.7]	98.2 [79.6–123.3]	0.014	84.6 [69.5–99.6]	100.9 [85.2–126.2]	0.004
≥80	81.4 [64.9–96.9]	90.0 [66.5–113.5]	0.055	81.4 [68.2–97.6]	93.4 [63.9–112.3]	0.268
All patients	86.5 [73.6–112.0]	102.7 [76.3–125.8]	<0.001	87.2 [72.9–112.4]	102.2 [76.1–124.3]	0.006

Values are the median follow-up in months [interquartile range], estimated by the reverse Kaplan–Meier method.
Group sizes for each stratum are given in Tables S2, S4, S6, and Table 4.
LLR, laparoscopic liver resection; OLR, open liver resection.

Table S2. Baseline characteristics of patients aged 65–69 years before and after propensity score matching

		Total	Before matching			After matching		
			LLR (n=108)	OLR (n=146)	P-value	LLR (n=71)	OLR (n=71)	P-value
Sex	Male	185	75	110	0.296	53	50	0.573
	Female	69	33	36		18	21	
BMI	<25 kg/m²	170	77	93	0.203	49	46	0.593
	≥25 kg/m²	84	31	53		22	25	
ECOG-PS	0	214	92	122	0.726	61	59	0.643
	≥1	40	16	24		10	12	
ASA	≤2	232	102	130	0.130	66	68	0.467
	≥3	22	6	16		5	3	
CCI	≤2	144	71	73	0.012	43	40	0.610
	≥3	110	37	73		28	31	
HBsAg	(+)	36	17	19	0.538	8	8	1.000
	(-)	218	91	127		63	63	
HCV Ab	(+)	116	48	68	0.736	37	32	0.401
	(-)	138	60	78		34	39	
LC	(+)	84	43	41	0.049	23	24	0.859
	(-)	170	65	105		48	47	
Child–Pugh score	5	200	81	119	0.438	54	53	0.799
	6	35	17	18		13	12	
	≥7	19	10	9		4	6	
Tumor diameter (cm)	≤3	183	86	97	0.021	54	55	0.843
	>3	71	22	49		17	16	
Tumor location	AL	170	86	84	<0.001	50	51	0.853
	PS	84	22	62		21	20	
Proximity to major vessels	(-)	218	100	118	0.008	65	65	1.000
	(+)	36	8	28		6	6	
History of upper abdominal surgery	(-)	225	94	131	0.505	62	63	0.796
	(+)	29	14	15		9	8	
Resected area	Wedge	111	72	39	<0.001	39	36	0.639
	Segment	47	9	38		9	8	
	Section	68	26	42		22	27	
	2 Sections	28	1	27		1	0	

AL, anterolateral; ASA, American Society of Anesthesiologists status; BMI, body mass index; CCI, Charlson Comorbidity Index; ECOG-PS, Eastern Cooperative Oncology Group performance status; HBsAg, hepatitis B surface antigen; HCV Ab, hepatitis C virus antibody; LC, liver cirrhosis; LLR, laparoscopic liver resection; OLR, open liver resection; PS, posterosuperior.

Table S3. Comparison of short-term outcomes between LLR and OLR in patients aged 65–69 years before and after propensity score matching

		Before matching			After matching		
		LLR (n=108)	OLR (n=146)	P-value	LLR (n=71)	OLR (n=71)	P-value
Blood loss (mL)	Median [range]	63 [0-2175]	390 [0-2590]	<0.001	78 [0-2175]	390 [0-2175]	<0.001
Blood transfusion	n (%)	3 (2.78)	12 (8.22)	0.104	1 (1.41)	7 (9.86)	0.063
Operation time (min)	Median [range]	263.5 [96-659]	339 [93-774]	<0.001	274 [121-659]	314 [93-528]	0.158
Margin (cm)	Median [range]	0.5 [0-7]	0.7 [0-12]	0.095	0.75 [0-7]	0.5 [0-12]	0.075
Postoperative complications							
Clavien–Dindo ≥2	n (%)	12 (11.11)	34 (23.29)	0.013	6 (8.45)	20 (28.17)	0.002
Mortality	n (%)	1 (0.93)	1 (0.68)	1.000	1 (1.41)	1 (1.41)	1.000
Postoperative hospital stay (days)	Median [range]	9 [4-103]	12 [7-114]	0.002	9 [4-35]	12 [7-66]	<0.001

LLR, laparoscopic liver resection; OLR, open liver resection.

Table S4. Baseline characteristics of patients aged 70–74 years before and after propensity score matching

		Total	Before matching			After matching		
			LLR (n=108)	OLR (n=144)	P-value	LLR (n=73)	OLR (n=73)	P-value
Sex	Male	166	67	99	0.266	47	44	0.608
	Female	86	41	45		26	29	
BMI	<25 kg/m²	174	71	103	0.325	53	51	0.715
	≥25 kg/m²	78	37	41		20	22	
ECOG-PS	0	191	85	106	0.350	54	55	0.849
	≥1	61	23	38		19	18	
ASA	≤2	231	101	130	0.357	68	68	1.000
	≥3	21	7	14		5	5	
CCI	≤2	130	63	67	0.064	38	38	1.000
	≥3	122	45	77		35	35	
HBsAg	(+)	36	12	24	0.275	9	8	0.796
	(-)	216	96	120		64	65	
HCV Ab	(+)	126	58	68	0.309	40	35	0.408
	(-)	126	50	76		33	38	
LC	(+)	92	44	48	0.227	29	29	1.000
	(-)	160	64	96		44	44	
Child–Pugh score	5	210	87	123	0.453	56	61	0.496
	6	32	17	15		13	8	
	≥7	10	4	6		4	4	
Tumor diameter (cm)	≤3	177	86	91	0.005	54	54	1.000
	>3	75	22	53		19	19	
Tumor location	AL	166	79	87	0.035	52	50	0.718
	PS	86	29	57		21	23	
Proximity to major vessels	(-)	222	103	119	0.002	68	70	0.467
	(+)	30	5	25		5	3	
History of upper abdominal surgery	(-)	209	91	118	0.628	61	60	0.826
	(+)	43	17	26		12	13	
Resected area	Wedge	108	68	40	<0.001	40	40	0.791
	Segment	46	11	35		11	12	
	Section	70	28	42		21	21	
	2 Sections	28	1	27		1	0	

AL, anterolateral; ASA, American Society of Anesthesiologists status; BMI, body mass index; CCI, Charlson Comorbidity Index; ECOG-PS, Eastern Cooperative Oncology Group performance status; HBsAg, hepatitis B surface antigen; HCV Ab, hepatitis C virus antibody; LC, liver cirrhosis; LLR, laparoscopic liver resection; OLR, open liver resection; PS, posterosuperior.

Table S5. Comparison of short-term outcomes between LLR and OLR in patients aged 70–74 years before and after propensity score matching

		Before matching			After matching		
		LLR (n=108)	OLR (n=144)	P-value	LLR (n=73)	OLR (n=73)	P-value
Blood loss (mL)	Median [range]	80 [0-1325]	374.5 [1-3920]	<0.001	93 [0-1325]	285 [1-2620]	<0.001
Blood transfusion	n (%)	4 (3.70)	12 (8.33)	0.192	4 (5.48)	3 (4.11)	1.000
Operation time (min)	Median [range]	231.5 [65-467]	335 [61-715]	<0.001	261 [65-467]	291 [61-590]	0.059
Margin (cm)	Median [range]	0.5 [0-4.5]	0.5 [0-10]	0.800	0.7 [0-4.2]	0.5 [0-4.5]	0.632
Postoperative complications							
Clavien–Dindo ≥2	n (%)	14 (12.96)	46 (31.94)	<0.001	10 (13.70)	23 (31.51)	0.010
Mortality	n (%)	0 (0.00)	0 (0.00)	1.000	0 (0.00)	0 (0.00)	1.000
Postoperative hospital stay (days)	Median [range]	10 [5-106]	15 [7-398]	0.002	10 [6-106]	14 [7-398]	0.053

LLR, laparoscopic liver resection; OLR, open liver resection.

Table S6. Baseline characteristics of patients aged 75–79 years before and after propensity score matching

		Total	Before matching			After matching		
			LLR (n=92)	OLR (n=175)	P-value	LLR (n=67)	OLR (n=67)	P-value
Sex	Male	175	60	115	0.935	48	46	0.706
	Female	92	32	60		19	21	
BMI	<25 kg/m²	184	67	117	0.317	45	49	0.450
	≥25 kg/m²	83	25	58		22	18	
ECOG-PS	0	206	69	137	0.543	50	48	0.697
	≥1	61	23	38		17	19	
ASA	≤2	243	82	161	0.436	58	62	0.259
	≥3	24	10	14		9	5	
CCI	≤2	145	50	95	0.992	35	37	0.729
	≥3	122	42	80		32	30	
HBsAg	(+)	23	8	15	0.973	7	6	0.770
	(-)	244	84	160		60	61	
HCV Ab	(+)	143	55	88	0.139	37	35	0.729
	(-)	124	37	87		30	32	
LC	(+)	74	31	43	0.113	20	18	0.702
	(-)	193	61	132		47	49	
Child–Pugh score	5	202	63	139	0.008	51	47	0.718
	6	51	19	32		14	18	
	≥7	14	10	4		2	2	
Tumor diameter (cm)	≤3	184	73	111	0.008	50	50	1.000
	>3	83	19	64		17	17	
Tumor location	AL	173	76	97	<0.001	52	53	0.834
	PS	94	16	78		15	14	
Proximity to major vessels	(-)	223	83	140	0.033	59	57	0.612
	(+)	44	9	35		8	10	
History of upper abdominal surgery	(-)	213	78	135	0.140	54	54	1.000
	(+)	54	14	40		13	13	
Resected area	Wedge	128	69	59	<0.001	44	44	0.943
	Segment	50	8	42		8	10	
	Section	65	13	52		13	11	
	2 Sections	24	2	22		2	2	

AL, anterolateral; ASA, American Society of Anesthesiologists status; BMI, body mass index; CCI, Charlson Comorbidity Index; ECOG-PS, Eastern Cooperative Oncology Group performance status; HBsAg, hepatitis B surface antigen; HCV Ab, hepatitis C virus antibody; LC, liver cirrhosis; LLR, laparoscopic liver resection; OLR, open liver resection; PS, posterosuperior.

Table S7. Comparison of short-term outcomes between LLR and OLR in patients aged 75–79 years before and after propensity score matching

		Before matching			After matching		
		LLR (n=92)	OLR (n=175)	P-value	LLR (n=67)	OLR (n=67)	P-value
Blood loss (mL)	Median [range]	100 [0-2933]	370 [3-2940]	<0.001	100 [0-1100]	295 [10-2250]	0.032
Blood transfusion	n (%)	4 (4.35)	20 (11.43)	0.042	3 (4.48)	4 (5.97)	0.698
Operation time (min)	Median [range]	228.5 [78-505]	321 [70-697]	<0.001	241 [99-505]	260 [70-697]	0.245
Margin (cm)	Median [range]	0.5 [0-5.5]	0.5 [0-6.5]	0.567	0.5 [0-5.5]	0.5 [0-5]	0.786
Postoperative complications							
Clavien–Dindo ≥2	n (%)	12 (13.04)	54 (30.86)	0.001	7 (10.45)	13 (19.40)	0.146
Mortality	n (%)	1 (1.09)	0 (0.00)	0.345	0 (0.00)	0 (0.00)	1.000
Postoperative hospital stay (days)	Median [range]	10 [6-56]	14 [3-96]	<0.001	10 [6-42]	12 [7-96]	0.046

LLR, laparoscopic liver resection; OLR, open liver resection.

Table S8. Comparison of short-term outcomes between LLR and OLR in patients aged ≥80 years before and after propensity score matching

		Before matching			After matching		
		LLR (n=85)	OLR (n=111)	P-value	LLR (n=54)	OLR (n=54)	P-value
Blood loss (mL)	Median [range]	50 [0-2657]	332 [10-2657]	<0.001	50 [0-1100]	255 [10-2250]	<0.001
Blood transfusion	n (%)	5 (5.88)	18 (16.22)	0.026	2 (3.7)	6 (11.1)	0.270
Operation time (min)	Median [range]	282 [67-722]	221 [65-647]	0.005	235 [95-565]	246 [65-647]	0.605
Margin (cm)	Median [range]	0.6 [0-3]	0.6 [0-5]	0.501	0.7 [0-3]	0.6 [0-5]	0.693
Postoperative complications							
Clavien–Dindo ≥2	n (%)	10 (11.76)	34 (30.63)	0.002	7 (12.96)	14 (25.93)	0.089
Mortality	n (%)	0 (0.00)	2 (1.89)	0.226	0 (0.00)	0 (0.00)	1.000
Postoperative hospital stay (days)	Median [range]	9 [5-74]	14 [5-73]	<0.001	9 [5-43]	13 [5-73]	0.009

LLR, laparoscopic liver resection; OLR, open liver resection.
